# Bee gomogenat rescues lymphoid organs from degeneration by regulating the crosstalk between apoptosis and autophagy in streptozotocin-induced diabetic mice

**DOI:** 10.1007/s11356-022-20457-x

**Published:** 2022-05-13

**Authors:** Gamal Badr, Leila H. Sayed, Hossam El-Din M. Omar, Sary Khaleel ِAbd Elghaffar, Medhat M. Menshawy

**Affiliations:** 1 Zoology Department, Faculty of Science, Assiut University, Assiut, 71516 Egypt; 2 Laboratory of Immunology, Zoology Department, Faculty of Science, Assiut University, Assiut, 71516 Egypt; 3 Pathology and clinical pathology Department, Faculty of Veterinary Medicine, Assiut University, Assiut, 71516 Egypt; 4 Department of Biology, Misr University of Science and Technology, 6th October City, Egypt

**Keywords:** Bee gomogenat, Diabetes mellitus, Cytokines, Oxidative Stress, Inflammation, Autophagy, Apoptosis

## Abstract

Diabetes mellitus (DM) is a metabolic disorder that causes severe complications in several tissues due to redox imbalances, which in turn cause defective angiogenesis in response to ischemia and activate a number of proinflammatory pathways. Our study aimed to investigate the effect of bee gomogenat (BG) dietary supplementation on the architecture of immune organs in a streptozotocin (STZ)-induced type 1 diabetes (T1D) mouse model. Three animal groups were used: the control non-diabetic, diabetic, and BG-treated diabetic groups. STZ-induced diabetes was associated with increased levels of blood glucose, ROS, and IL-6 and decreased levels of IL-2, IL-7, IL-4, and GSH. Moreover, diabetic mice showed alterations in the expression of autophagy markers (LC3, Beclin-1, and P62) and apoptosis markers (Bcl-2 and Bax) in the thymus, spleen, and lymph nodes. Most importantly, the phosphorylation level of AKT (a promoter of cell survival) was significantly decreased, but the expression levels of MCP-1 and HSP-70 (markers of inflammation) were significantly increased in the spleen and lymph nodes in diabetic mice compared to control animals. Interestingly, oral supplementation with BG restored the levels of blood glucose, ROS, IL-6, IL-2, IL-4, IL-7, and GSH in diabetic mice. Treatment with BG significantly abrogated apoptosis and autophagy in lymphoid organs in diabetic mice by restoring the expression levels of LC3, Beclin-1, P62, Bcl-2, and Bax; decreasing inflammatory signals by downregulating the expression of MCP-1 and HSP-70; and promoting cell survival by enhancing the phosphorylation of AKT. Our data were the first to reveal the therapeutic potential of BG on the architecture of lymphoid organs and enhancing the immune system during T1D.

## Introduction

Diabetes mellitus (DM) is a metabolic disorder that causes hyperglycemia due to relative or absolute insulin deficiency (King 2012). Furthermore, type 1 diabetes (TID) causes disturbances in T- and B-cell functions, leading to an increase in infections (Mikulkova et al. 2010; Giese et al. 2021). T1D patients show high morbidity and mortality rates as a result of increased susceptibility to infections (Casqueiro et al. 2012). Moreover, proinflammatory cytokines are secreted by monocytes in diabetic patients, leading to alterations in immune cell responses (Khumaedi et al. 2019). In DM, hyperglycemia induces oxidative stress, and glucose toxicity due to autoxidation is thought to be one of the major sources of ROS (Al-Hariri et al. 2011). In this context, during diabetes, ROS accumulation leads to increases in free radicals and excessive inflammation, which are the major factors that contribute to disturbed organ functions (He et al. 2017). Oxidative stress is the state that arises from an imbalance between ROS formation and the ability of the antioxidant system to neutralize these compounds (Pizzino et al. 2017). ROS destroy different organs through the oxidation of DNA, proteins, and intracellular macromolecules and peroxidation of membrane lipids. Changes in biomarkers of oxidative stress, including glutathione (GSH), catalase, glutathione peroxidase (GSH Px), superoxide dismutase (SOD), GSH reductase, and certain related genes, could be used to quantify oxidative damage in diabetes (Das and Sil 2012).

Cytokines are a group of glycoproteins that modulate the activity of individual cells under both pathological and physiological conditions (Tanaka et al. 2014). Additionally, cytokines are important mediators of cell functions in normal and disease states (Turner et al. 2014). Proinflammatory cytokines, such as interleukin-6 (IL-6), are pleiotropic factors that regulate immune and inflammatory reactions (Banerjee and Saxena 2012; Schett 2018). In the periphery, it has been shown that the levels of IL-2 and IL-7 maintain the survival and function of mature T cells (Davidsson et al. 2013; Chen et al. 2021), while IL-4 plays a key role in the activation of B cells; hence, IL-2, IL-7, and IL-4 are very important for the activation of adaptive immunity (Granato et al. 2014). Heat shock protein 70 (HSP-70) is an ATP-dependent chaperone in the cytosol that is part of a group of autoantigens with the ability to stimulate immunoregulatory pathways during inflammatory diseases in humans, such as T1D, rheumatoid arthritis (RA), and atherosclerosis (Tukaj 2020). The expression of HSP-70 is increased due to cellular stress, leading to effective protection against damaging mediators in islets β cells isolated from rats and humans (Lee et al. 2013). Monocyte chemoattractant protein-1 (MCP-1) is a strong chemokine that is capable of stimulating leucocytes, particularly macrophages and monocytes. A recent study demonstrated that MCP-1 plays an important role in the inflammatory process in diabetic nephropathy (Yap 2017).

Autophagy and apoptosis are two catabolic pathways that are necessary for organismal homeostasis and development (Su et al. 2013; Nikoletopoulou et al., 2013; Fairlie et al. 2020; Sareen et al., 2022). Autophagy is a cell survival pathway that regulates cell death under certain conditions and is mediated by several proteins, such as P62 (also known as sequestosome-1), Beclin-1, and microtubule-associated protein light chain 3 (LC3) (Su et al. 2013; Xu et al. 2013; Li et al. 2016). Apoptosis is a form of programmed cell death and is caused by many antiapoptotic proteins, such as B-cell lymphoma 2 (Bcl-2), and proapoptotic proteins, such as Bcl-2-associated X protein (Bax) (Su et al. 2013; Kouri et al. 2012).

Recently, researchers have paid more attention to diabetes and its associated complications. Thus, novel and more effective therapeutic agents for treating diabetic complications are urgently needed and should be developed.

Bee gomogenat (BG) of trutnevy larvae contains a mostly nutritious mixture consisting of proteins, vitamins (A, B, E, D, B-carotene), amino acids, minerals (magnesium, potassium, calcium, phosphorus, iron, zinc), various enzymes, and steroid hormones (an estradiol, progesterone). However, there have been no published papers concerning the biological effects of BG on animal models. Hence, we investigated the effect of BG on different animal models, including heat stress, diabetes and gastric ulcers. Our preliminary results demonstrated the potential therapeutic effect of BG. However, our study aimed to investigate the impact of dietary supplementation with BG on oxidative stress, inflammation, immune cells, and architectures of lymphoid organs in a streptozotocin (STZ)-induced T1D mouse model and clarify the underlying molecular mechanisms.

## Materials and Methods

### Bee gomogenat preparation

BG was purchased from Etman Hives for Honeybee Products (Tanta, Egypt). The active chemical constituents of BG were analyzed by GC–MS (The Analytical Chemistry Unit of the Chemistry Department, Faculty of Science, Assiut University). BG is a creamy substance when stored at − 20 °C. Based on our preliminary experiments, we found no adverse effects of high-dose oral BG supplementation on mice up to 4 g/kg body weight, and the optimal dose was 1 g/kg body weight. In the current study, the optimal dose of BG that was prepared by dissolving 1 g of BG in a final volume of 10 ml of distilled H_2_O (100 mg/ml). Then, 250 μl of diluted BG (25 mg) was orally administered to each mouse weighing 25 g (1 g/kg body weight/day for 30 days).

#### Chemicals

STZ was purchased from Sigma Chemicals Co. (St. Louis, MO, USA). STZ was freshly prepared for immediate use (within 5 min) by dissolving in cold 0.01 M citrate buffer (pH 4.50).

#### Experimental design and treatments

Forty-five BALB/c adult male mice (25–30 g) were obtained from the Institute of Theodor Bilharz (Cairo, Egypt). The mice were housed in cages at 25 ± 5 °C under a normal 12 h light/12 h dark cycle. The mice were fed a grain- and water-based diet for 1 week to acclimatize. All animal experiments were carried out according to the Institutional Animal Care laws and the International Guidelines for Animal Care (Council of European Communities 1986) and were approved by the Ethics Committee of the Faculty of Medicine at Assiut University (Ethics approval number 163/2204-2020). We minimized animal distress and kept their number to a minimum as previously described (Al Ghamdi et al. 2015). The mice were divided into three groups of 15 mice each after 1 week of acclimatization: non-diabetic control (cont.), diabetic (diab.), and diabetic plus BG (diab. + BG). Diabetes was induced in mice in groups 2 and 3 by three intraperitoneal injections (i.p.) of STZ (60 mg/kg body weight) in 0.01 M citrate buffer (pH 4.5). Mice were considered diabetic if their glycemia level exceeded 220 mg/dl. Control group mice were injected with the vehicle (0.01 M citrate buffer, pH 4.5). After 2 weeks of intraperitoneal injection with STZ, control non-diabetic mice were orally supplemented with distilled water (250 μl/mouse/day for one month by oral gavage), group 2 diabetic mice were orally supplemented with distilled water (250 μl/mouse/day for one month by oral gavage), and group 3 diabetic mice were orally supplemented with BG (1 g/kg body weight/day for one month by oral gavage).

#### Blood collection and analysis

Blood was drawn from the abdominal aorta and immediately placed into EDTA tubes. To collect plasma and eliminate red blood cells, the blood samples were centrifuged at 5000 g for 10 min using a cooling centrifuge (Anke TGL-16B). Dry Pasteur pipettes were used to collect plasma samples, which were then stored at − 20 °C until needed.

#### Measuring ROS levels

The 2,7-dichlorodihydrofluorescein diacetate (H2DCF-DA) method was used to assess ROS levels in plasma (Beyotime Institute of Biotechnology, Haimen, China). To quantify H_2_O_2_ levels, the oxidation of 2′-7′ dichlorofluorescin (H2DCF) to 2′-7′-dichlorofluorescein (DCF) was examined. H2DCF is oxidized by ROS and converted to 2′,7′ dichlorodihydrofluorescein (DCF), a highly fluorescent compound. The excitation and emission wavelengths used to assess DCF fluorescence were 498 nm and 522 nm, respectively (Sayed et al. 2012)

#### Measuring cytokine levels

The plasma cytokine profile was evaluated in samples that were stored at − 80 °C. The cytokine levels (IL-6, IL-2, IL-4, and IL-7) were determined by ELISA using a Bio-Plex Mouse Cytokine Assay Kit (Bio–Rad, Hercules, CA, USA) according to the manufacturer’s instructions as previously described (Ramadan et al. 2021).

#### Glutathione and GSH Px assay

Plasma was subjected to a GSH assay kit (Cayman Chemical, # 703002, Ann Arbor, MI, USA) and GSH peroxidase assay kit (Abcam ab102530, USA) according to the manufacturer’s instructions as previously described (Sayed et al. 2017).

#### MnSOD and catalase activity assay

Manganese superoxide dismutase (MnSOD) activity was assessed in plasma. Briefly, aliquots of plasma were treated with a CuZnSOD inhibitor and were then subjected to a commercial SOD assay kit (Cayman Chemical, # 706002, Ann Arbor, MI, USA) according to the manufacturer’s instructions. Catalase activity in plasma was evaluated using a commercial catalase activity assay kit (Cayman Chemical, no. 707002, Ann Arbor, MI, USA) according to the manufacturer’s instructions as previously described (Hozzein et al. 2018).

#### Western blot analysis

RIPA buffer was used to prepare lysates from the tissues of the immune organs. Protein concentrations were measured using a protein assay kit (Bio–Rad, Hercules, CA). Forty micrograms of protein lysate was separated by SDS–PAGE prior to being transferred onto nitrocellulose membranes. The membranes were then blocked for 1 h using nonfat milk (50 g/L) in TBS, after which they were incubated overnight with primary antibodies against LC3, Beclin-1, P62, Bcl-2, Bax, and β-actin (1:1000; Santa Cruz Biotechnology). Then, HRP-conjugated species-matched secondary antibodies were used, the protein bands were detected with enhanced chemiluminescence (ECL, Super Signal West Pico Chemiluminescence Substrate, Perbio, Bezons, France), and the ECL signals were recorded using a LI-COR scanner. ImageJ software was used to quantify the protein band intensities as previously described (Badr and Mohany 2011; Badr et al. 2021).

#### Histopathological and immunohistochemical analysis

Thymus, liver, spleen, and lymph node samples were fixed immediately in formal alcohol until being processed as previously described (Mohany et al. 2011). The samples were then dehydrated and embedded, and thin sections (5 μm) were prepared. For histopathological examination, the sections were stained with H&E and Sirius red. For immunohistochemistry, tissue sections were processed according to Ramadan et al. (2018). We stained the tissue sections with the following primary antibodies: anti-CD4 or anti-CD19, anti-HSP-70, anti-MCP-1, and anti-AKT (Santa Cruz Biotechnology).

#### Statistical analysis

Statistical analysis was performed based on normally distributed data, which are expressed as the means ± standard error of the mean (SEM) using GraphPad Prism software version 5. Significant differences between the three groups were analyzed using one-way ANOVA followed by Tukey’s posttest.

## Results

### Analysis of the chemical composition of BG using GC–MS

**Fig. 1:**
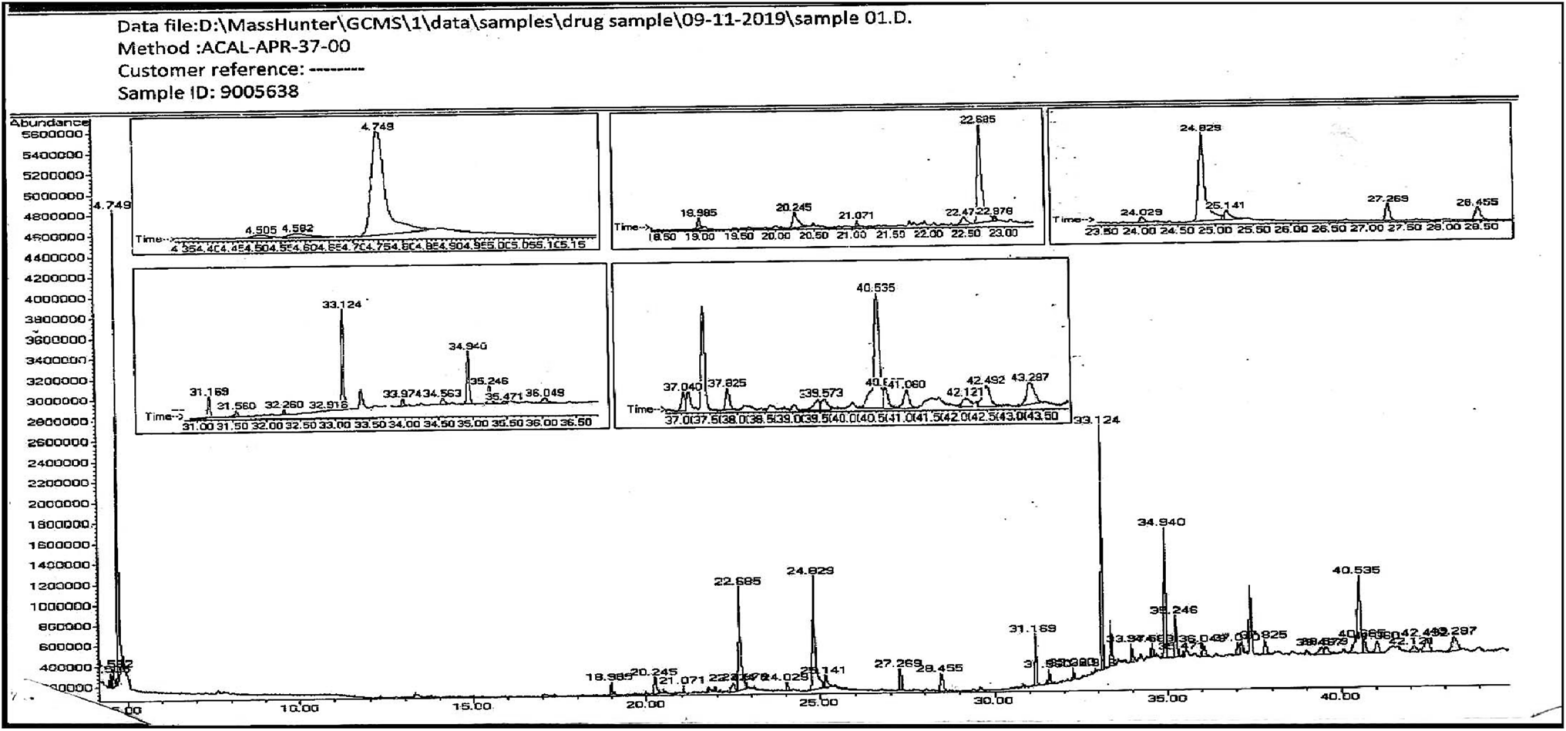
GC-MS results of the BG extract

The retention time (RT) of each compound was determined and is shown in Fig. 1. Twenty-seven compounds were identified in the BG extract (Table 1). The chemical constituents of BG are shown in Table 1 and are as follows: heptane (RT = 4.51 min, .53%), hexanone (RT = 4.58 min, 1.13%), octane (RT = 4.75 min, 17.67%), 1-tetradecanol (RT = 18.98 min, 0.38), tetradecanoic acid (RT = 20.25 min, 1.30%), hexadecanoic acid (RT = 22.68 min, 7.02%), *n*-pentacos-3-ene (RT = 22.88 min, 0.2%), triacontane (RT = 24.03 min, 0.47%), E-9-octadecenoic acid (RT = 24.83 min, 10.22%), (Z) -9-octadecenoic acid (RT = 25.14 min, 1.22%), nonadecane (RT = 27.27 min, 1.29%), 4,8,12,16-tetramethylheptadecan-4-olide (RT = 28.45 min, 1.43%), docosane (RT = 31.17 min, 2.25%), 1,22-dibromodocosane (RT = 31.65 min, 0.77%), heptadecane (RT = 32.26 min, 0.39%), oleyl alcohol heptafluorobutyrate (RT = 32.92 min, 0.14%), 2,6,10,14-tetramethylhexadecane (RT = 33.13 min, 7.79%), tetracosane (RT = 34.94 min, 5.27%), octadecane (RT = 35.25 min, 2.28%), 22-tricosenoic acid (RT = 35.47 min, 0.33%), octadecanoic acid, 2-(octadecyloxy) ethyl ester (RT = 36.06 min, 0.96%), (Z)-13-docosenoic acid (RT = 37.04 min, 0.89%), hexadecanoic acid, 2-(octadecyloxy)ethyl ester (RT = 37.83 min, 1.16%), 6Z-6-octadecenoic acid (RT = 39.46 min, 0.87%), heptafluorobutanoic acid, heptadecyl ester (RT = 40.54 min, 9.62%), octadecanoic acid (RT = 41.06 min, 1.32%), and trans-13-octadecenoic acid (RT = 42.12 min, 0.83%).

**Table 1:** The most abundant compound probabilities in BG using GC-MS. The GC-MS analysis of BG showing the identified compounds. *RT* retention time per minute; active compounds were detected by GC mass, *area (%)* percentage of compound, *M. formula* molecular formula, *M. wt* molecular weight of the compound

No.	RT (min)	Compound	Area (%)	M. formula	M. wt
1	4.51	Heptane	0.53	C_7_H_16_	100.21
2	4.58	Hexanone	1.13	C_6_H_12_O	100.16
3	4.75	Octane	17.67	C_8_H_18_	114.23
4	18.98	1-tetradecanol	0.38	C_14_H_30_O	214.39
5	20.25	Tetradecanoic acid	1.30	C_14_H_28_O_2_	229.36
6	22.68	Hexadecanoic acid	7.02	C_16_H_32_O_2_	256.42
7	22.88	*n*-pentacos-3-ene	0.20	C25H50	350.66
8	24.03	Triacontane	0.47	C_30_H_62_	422.82
9	24.83	E-9-octadecenoic acid 9-octadecenoic acid (E)	10.22	C_18_H_34_O_2_	282.46
10	25.14	(Z)-9-octadecenoic acid (Z)-octadec-9-enoic acid	1.22	C_18_ H_34_ O_2_	282.46
11	27.27	Nonadecane	1.29	C_19_H_40_	268.52
12	28.45	4,8,12,16-tetramethylheptadecan-4-olide	1.43	C_21_H_40_O_2_	324.54
13	31.17	Docosane	2.25	C_22_H_46_	310.60
14	31.56	1,22-dibromodocosane	0.77	C_22_H_44_Br_2_	468.39
15	32.26	Heptadecane	0.39	C_17_H_36_	240.47
16	32.92	Oleyl alcohol heptafluorobutyrate	0.14	C_22_H_35_F_7_O_2_	464.50
17	33.13	2,6,10,14-tetramethylhexadecane	7.79	C_20_H_42_	282.55
18	34.94	Tetracosane	5.27	C_24_H_50_	338.65
19	35.25	Octadecane	2.28	C_18_H_38_	254.49
20	35.47	22-tricosenoic acid	0.33	C_23_H_44_O_2_	352.59
21	36.06	Octadecanoic acid, 2-(octadecyloxy)ethyl ester	0.96	C_38_H_76_O_3_	580
22	37.04	(Z)-13-docosenoic acid 13-cis-docosenoic acid	0.89	C_22_H_42_O_2_	338.57
23	37.83	Hexadecanoic acid, 2-(octadecyloxy)ethyl ester	1.16	C_36_H_72_O_3_	552.95
24	39.46	6Z-6-octadecenoic acid (6*Z*)-octadec-6-enoic acid	0.87	C_18_H_34_O	282.46
25	40.54	Heptafluorobutanoic acid, heptadecyl ester	9.62	C_21_H_35_F_7_O_2_	452.49
26	41.06	Octadecanoic acid	1.32	C_18_H_36_O_2_	285.47
27	42.12	Trans-13-octadecenoic acid	0.83	C_18_H_34_O_2_	282.46

### The biological activities of the compounds isolated from BG

According to the GC–MS results, most of the identified compounds possess interesting biological activities, as shown in Table 2. Among the identified chemicals, *n*-hexadecanoic acid has anti-inflammatory properties (Ghaidaa et al. 2016). Octadecenoic acid has antibacterial activity (Agoramoorthy et al. 2007). Tetracosane has effective antioxidant activity (Lomarat et al. 2015). Additionally, 1-tetradecanol has both antibacterial and anti-inflammatory activities (Geng et al. 2018). Moreover, octadecane has both antimicrobial and antioxidant activities (Mishra and Shree 2007; Lee et al. 2007). Docosane has antimicrobial activity (Lammers et al. 2021). Heptadecane has antimicrobial, antioxidant (Mishra and Shree 2007; Lee et al. 2007), and anti-inflammatory activities (Kim et al. 2013). Finally, nonadecane has antioxidant, antibacterial (Javidnia et al. 2008), and antimicrobial activities (Mahmoodreza et al. 2010).

**Table 2:** Most abundant compounds with biological activities

No.	Compound	Biological activity	References
1	*n*-hexadecanoic acid	Anti-inflammatory	Ghaidaa et al. (2016)
2	Octadecenoic acid	Antibacterial activity	Agoramoorthy et al. (2007)
3	Tetracosane	Antioxidant activity	Lomarat et al. (2015)
4	1-tetradecanol	Antibacterial and anti-inflammatory	Geng et al. (2018)
5	Octadecane	Antimicrobial and antioxidant	Mishra and Shree (2007); Lee et al. (2007)
6	Docosane	Antimicrobial activity	Lammers et al. (2021)
7	Heptadecane	Antimicrobial and antioxidant Anti-inflammatory	Mishra and Shree (2007); Lee et al. (2007); Kim et al. (2013)
8	Nonadecane	Antioxidant, antibacterial antimicrobial	Javidnia et al. (2008); Mahmoodreza et al. (2010)

### Supplementation with BG decreases blood glucose levels and restores body weight in diabetic mice

Throughout the investigation, we tracked changes in blood glucose levels and body weight in all groups. When compared to that of control mice, STZ caused substantial hyperglycemia, as evidenced by significant increases in blood glucose levels and reductions in body weight (*n* = 5, *P 0.05) (Table 3). BG treatment of diabetic animals restored blood glucose levels and body weight compared to those of untreated diabetic animals

**Table 3:** Influence of BG on blood glucose levels and body weight in diabetic mice. Body weight and blood glucose levels were evaluated in three groups of mice throughout the experimental period. The accumulated data from five mice in each group are expressed as the mean value for each parameter ± SEM.

Groups	Glucose level (mg/dl)	Body weight (g)
Cont.	108.7 ± 4.53	29.29 ± 0.95
Diab.	415.6 ± 50.50*	23.17 ± 0.59*
Diab.+BG	273.8 ± 36.15**#+**	24.41 ± 0.79**+**

*P < 0.05 for diab. vs. cont.; +*P* < 0.05 for diab.+BG vs. cont.; #*P* < 0.05 diab.+BG vs. diab. (ANOVA with Tukey’s post-test)

### BG supplementation decreases free radical and proinflammatory cytokine levels and increases IL-2, IL-4, and IL-7 levels; GSH levels; and antioxidant enzyme activities in diabetic mice

**Fig. 2:**
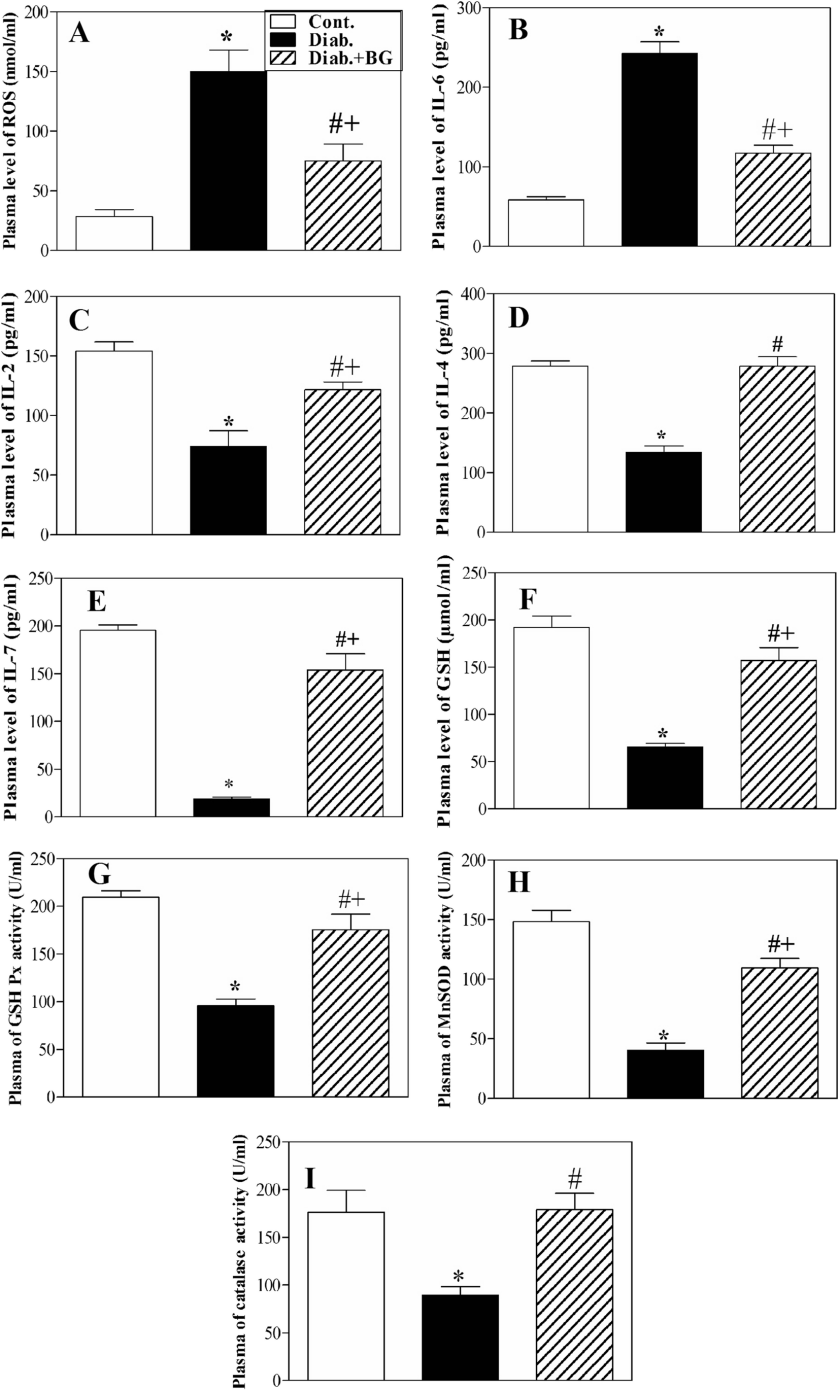
Effect of BG on the levels of ROS, IL-6, IL-2, IL-4, IL-7 and GSH and on the activities of antioxidant enzymes in mice with T1D. The levels of ROS (**A**), IL-6 (**B**), IL-2 (**C**), IL-4 (**D**), IL-7 (**E**), and GSH (**F**) and the activities of GSH Px (**G**), MnSOD (**H**), and catalase (**I**) were measured in the plasma of cont. (open bars), diab. (closed black bars), and diab.+BG. (hatched bars) animals. Accumulated data from five mice in each group are expressed as the mean ± SEM (*n* = 5). ^*^P < 0.05 for diab. vs. cont.; ^+^*P* < 0.05 for diab.+BG vs. cont.; and ^#^*P* < 0.05 diab.+BG vs. diab. (ANOVA with Tukey’s post-test)

We evaluated the levels of ROS, IL-6 (a proinflammatory cytokine), IL-2 and IL-7 (important cytokines for the survival of T cells), and IL-4 (B-cell activation), GSH, and the activities of antioxidant enzymes (GSH Px, MnSOD and catalase) in plasma. The data are expressed as the value ± SEM (Fig. 2). The plasma levels of ROS (Fig. 2A) and IL-6 (Fig. 2B) were significantly higher in the diabetic mice than in the control non-diabetic mice (*P < 0.05). Interestingly, compared to diabetic mice, BG-treated diabetic mice exhibited significant restoration of ROS and IL-6 levels (#*P* < 0.05), while diabetic mice exhibited decreased levels of IL-2 (Fig. 2C), IL-4 (Fig. 2D), and IL-7 (Fig. 2E) compared to control non-diabetic animals. Furthermore, compared to untreated diabetic mice, BG-treated diabetic mice displayed significant restoration of IL-2, IL-4, and IL-7 levels (^#^*P* < 0.05). Additionally, the levels of GSH significantly (^*^P < 0.05) decreased in diabetic mice compared to control non-diabetic mice (Fig. 2F). When diabetic mice were supplemented with BG, they exhibited significant (^#^*P* < 0.05) elevations in the levels of GSH compared to distilled water-treated diabetic mice. Likewise, the activities of GSH Px (Fig. 2G), MnSOD (Fig. 2H), and catalase (Fig. 2I) were significantly (^*^P < 0.05) decreased in diabetic mice compared to control non-diabetic mice. Most interestingly, treatment of diabetic mice with BG significantly (^#^*P* < 0.05) restored the activities of GSH Px, MnSOD, and catalase compared to those in diabetic mice.

### Supplementation with BG blunts pathological alterations and improves the altered distribution of T cells in the thymus in diabetic mice

**Fig. 3:**
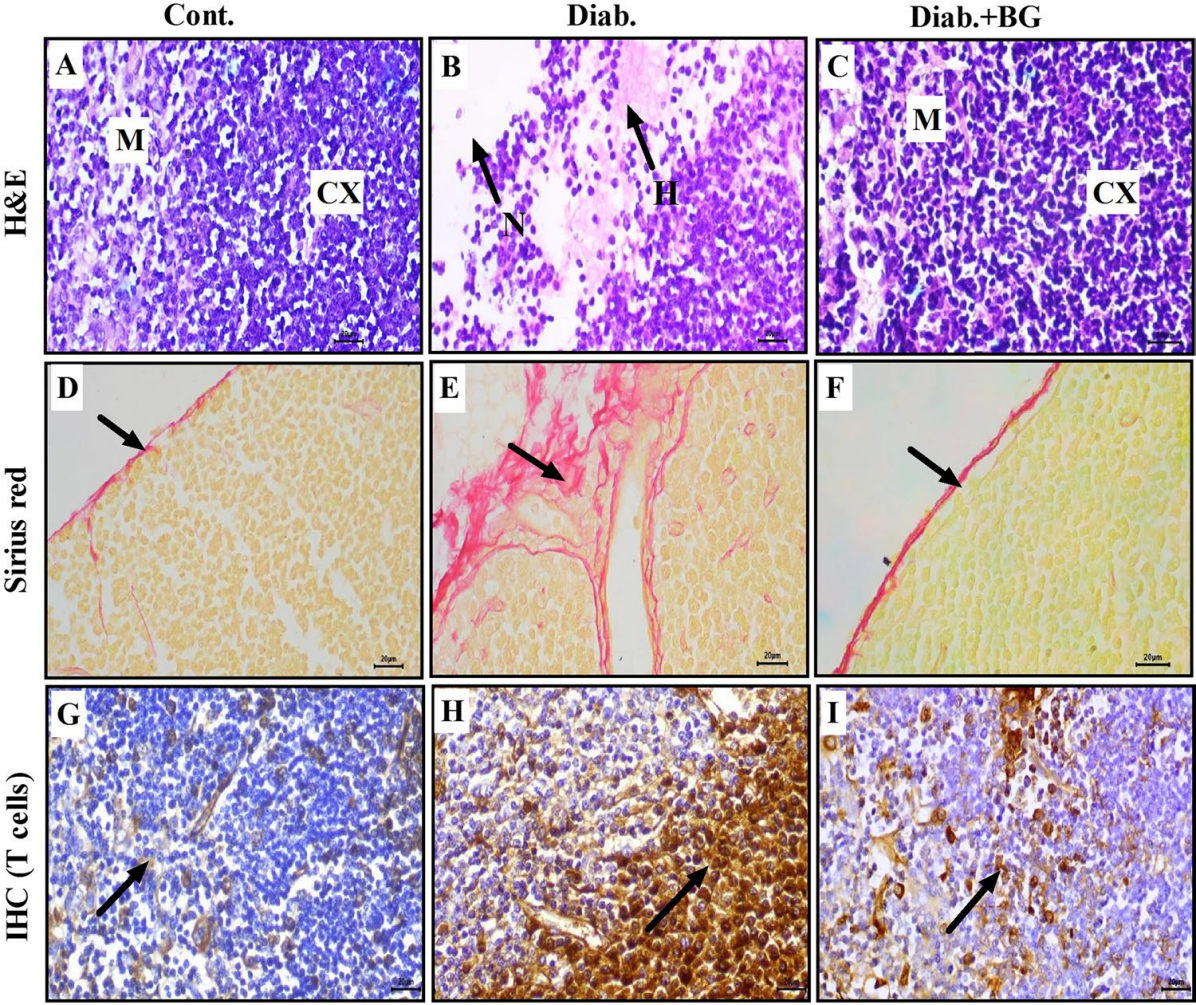
Oral supplementation with BG boosted the histological structure and distribution of T cells in the thymus during T1D. Histological changes in the thymus were assessed by H&E, Sirius red, and immunohistochemical staining. Photomicrographs of thymus sections from the cont., diab., and diab.+BG groups were stained with H&E (**A**–**C**), Sirius red to analyze the deposition of collagen (**D**–**F**), and immunohistochemically with anti-CD4 (**G**–**I**). (× 400)

We examined the histopathological changes in the thymus (as a primary immune organ) in the three animal groups after diabetes induction with STZ using H&E, Sirius red, and immunohistochemical (IHC) staining techniques. Pictures of H&E-stained tissue in the control, diabetic, and Diab.+BG groups were taken at × 400 magnification, and photographs of one representative are displayed. The thymus sections in the control group revealed normal histological appearance of the cortex and medulla (Fig. 3A). However, thymus sections in the diabetic group displayed degenerative changes represented by necrosis and hemorrhage in the cortex and medulla (Fig. 3B). The Diab.+BG group showed partial restoration of histological architecture in the thymus, similar to that of control mice (Fig. 3C). The deposition of collagen fibers in the thymus in diabetic animals was monitored using the Sirius red staining. Photomicrographs of Sirius red-stained thymus sections from the three groups were taken at × 400 magnification. Sirius red staining revealed normal distribution of collagenous fibers localized in the thymic capsule (Fig. 3D). In diabetic mice, Sirius red staining demonstrated a marked increase in collagenous fibers in the thymic capsule (Fig. 3E). Most importantly, treatment of the diabetic group with BG resulted in moderate amounts of collagenous fibers in the thymic capsule, similar to that in the control group (Fig. 3F). We investigated the effects of diabetes on the distribution of thymic T-lymphocytes in the three animal groups using anti-CD4. The thymus sections showed a normal distribution of T cells in the medulla in control animals (Fig. 3G), while diabetic animals exhibited an obvious increase in the number of T cells in the medulla (Fig. 3H). BG-treated diabetic mice displayed a partial restoration of T-cell distribution in the medulla, similar to that observed in control mice (Fig. 3I).

### BG supplementation repairs pathological damage by restoring the expression of MCP-1 in liver sections from diabetic mice

**Fig. 4:**
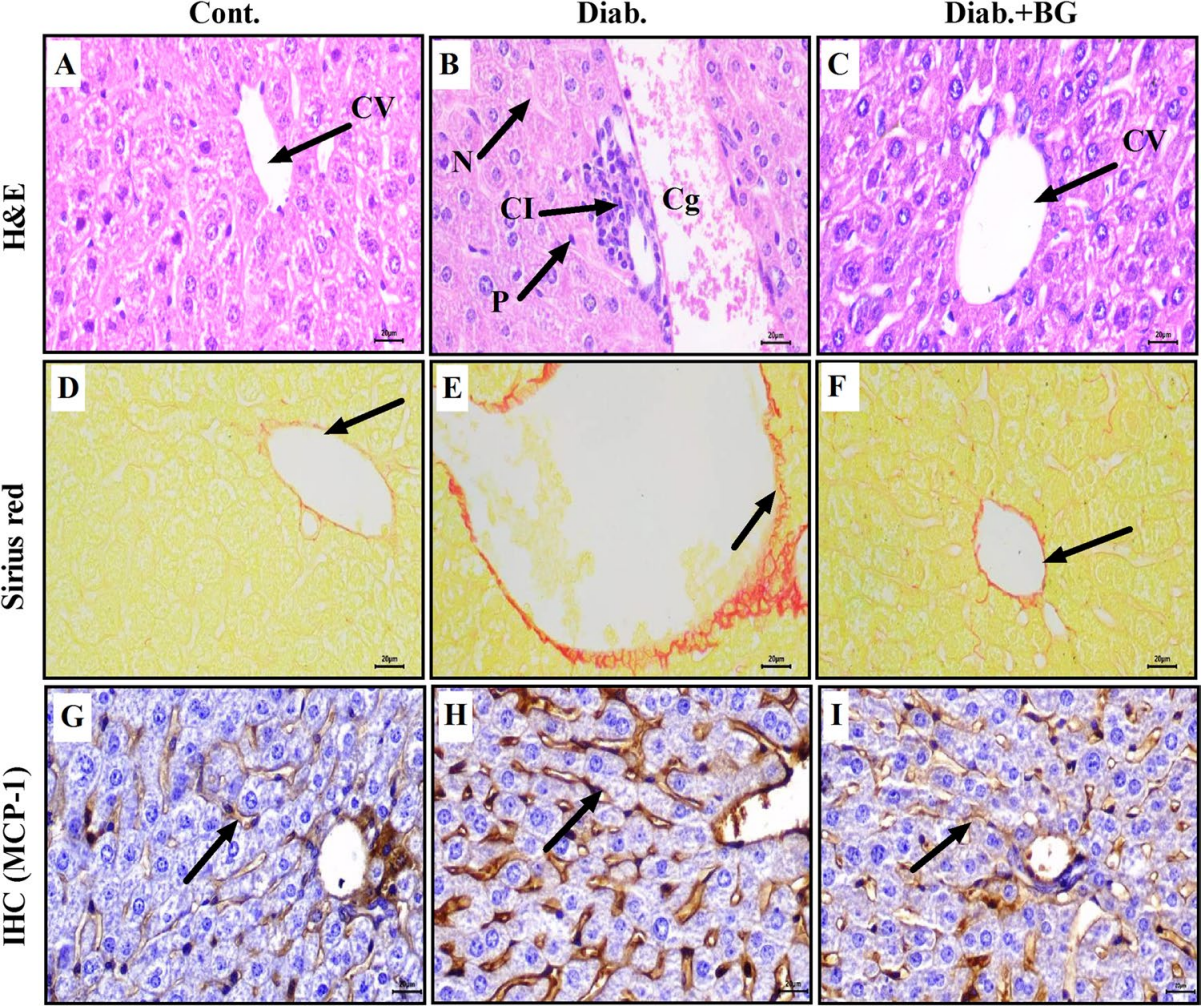
Impact of BG on architecture and the expression of MCP-1 in liver sections. Liver sections from the three groups were assessed by H&E, Sirius red, and immunohistochemical staining. Photomicrographs of liver sections in the cont., diab., and diab.+BG groups were stained with H&E to investigate histopathological changes (**A**–**C**) and Sirius red to examine the deposition of collagen (**D**–**F**) and examined by immunohistochemistry with anti-MCP-1 (**G**–**I**). (× 400)

The liver sections from the three groups showed histological alterations when examined by H&E, Sirius red, and IHC staining methods. Pictures of H&E-stained tissue in the control (Fig. 4A), diabetic (Fig. 4B), and Diab.+BG (Fig. 4C) groups were taken at × 400 magnification, and one representative photograph from each group is shown. The liver sections of control animals exhibited normal histological structures in the liver, with normal central veins and hepatic cords and hepatocytes (Fig. 4A). However, the liver sections of diabetic animals showed marked congestion of the blood vessels in the portal triad, and few hepatic cells underwent necrosis, which was associated with cellular infiltration and pyknotic nuclei in hepatic cells (Fig. 4B). The histological structure of the liver in the Diab.+BG group showed normal central veins, hepatic cords, and hepatocytes (Fig. 4C). One representative photomicrograph shows liver sections from the three groups were stained with Sirius red, and images were taken at × 400 magnification. The results revealed normal collagenous fibers around the central vein in the liver sections (Fig. 4D). However, in diabetic mice, Sirius red staining demonstrated a marked increase in collagenous fibers around the central vein (Fig. 4E) compared to those in the control group. Interestingly, treatment of diabetic mice with BG resulted in a marked decrease in the deposition of collagenous fibers around the central vein (Fig. 4F), similar to that in the control. We investigated the effect of diabetes on the expression of MCP-1 in liver sections in the three groups using anti-MCP-1 as a marker of inflammation. At × 400 magnification, the liver sections showed normal expression of MCP-1 in the endothelial cells lining the blood sinusoids of the liver in control mice (Fig. 4G), while the diabetic group showed a marked increase in the expression of MCP-1 in endothelial cells lining the blood sinusoids of the liver (Fig. 4H). In BG-treated diabetic mice, partial restoration in the expression of MCP-1 in endothelial cells lining blood sinusoids was similar to the results seen in control mice (Fig. 4I).

### BG reduces histopathological alterations and enhances the distribution of T and B cells, the expression level of HSP-70, and the phosphorylation level of AKT in the spleens of diabetic mice

**Fig. 5:**
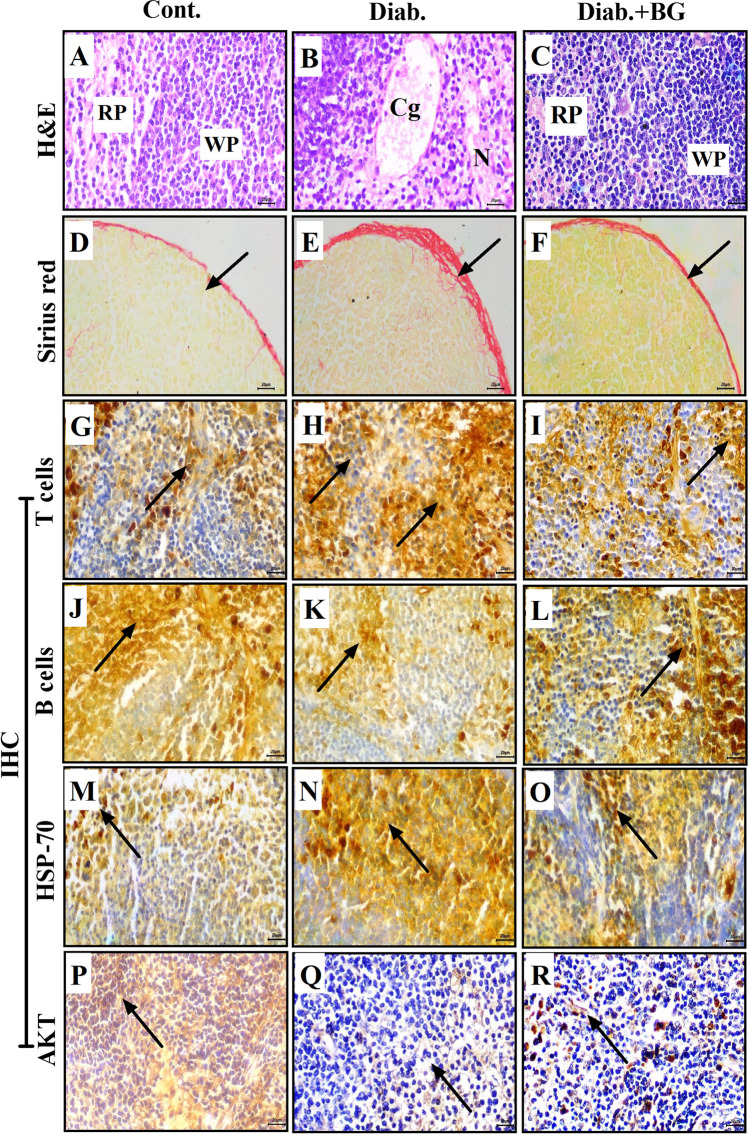
Impact of BG supplementation on the distribution of T and B cells and on the expression of HSP-70 and the phosphorylation of AKT in the spleens of diabetic mice. Sections of spleen from three groups were assessed by H&E, Sirius red, and immunohistochemical staining. Photomicrographs of spleen sections in the cont., diab., and diab.+BG groups were stained with H&E to examine histopathological changes (**A–C**), Sirius red (**D**–**F**), and examined by immunohistochemistry with anti-CD4 (**G–I**), anti-CD19 (**J–L**), anti-HSP-70 (**M–O**), and anti-AKT (**P**–**R**). (× 400)

Histopathological alterations were monitored in the spleen (as a secondary immune organ) in the three animal groups after the induction of diabetes using H&E, Sirius red, and IHC staining. H&E staining was performed, a micrograph at × 400 magnification was taken from the control, diabetic, and Diab.+BG groups, and one representative image from each group is shown. Spleen sections from control animals showed a normal histological appearance with white pulp, which was composed of condensations of lymphocytes around the central artery, and red pulp, which was composed of splenic cords and sinuses (Fig. 5A). However, spleen sections from diabetic animals exhibited degenerative changes, as represented by necrosis and congestion in red and white pulp (Fig. 5B). The Diab.+BG group showed partially restored histological architecture in spleen tissue similar to that of control mice (Fig. 5C). Sirius red staining was performed, and photomicrographs of spleen sections from the three groups were taken at × 400 magnification. The results demonstrated normal distribution of collagenous fibers in the splenic capsule (Fig. 5D). Diabetic animals exhibited a marked increase in collagenous fibers in the splenic capsule (Fig. 5E). Interestingly, treatment of diabetic mice with BG decreased the amounts of collagenous fibers in the splenic capsule to levels similar to those in the control group (Fig. 5F). At × 400 magnification, we investigated the effect of diabetes on the distribution of T cells and B cells in the spleen using anti-CD4 and anti-CD19 antibodies. The spleen sections showed a normal number of T cells in the periarterial lymphatic sheath of white and red pulp of the control group (Fig. 5G), while diabetic mice had increased numbers of T cells in the periarterial lymphatic sheath of white and red pulp (Fig. 5H). BG-treated diabetic mice showed partially restored T-cell distribution in the periarterial lymphatic sheath of white and red pulp similar to that observed in control mice (Fig. 5I). The spleen sections of control mice showed a normal number of B cells in the lymphoid follicles of white pulp (Fig. 5J), whereas in diabetic animals, the number of B cells was decreased in the lymphoid follicles of white pulp (Fig. 5K). Supplementation of diabetic mice with BG restored the distribution of B cells in the lymphoid follicles of white pulp nearly to control levels (Fig. 5L). Additionally, we investigated the expression of HSP-70 (as a marker of inflammation) in spleen sections in the three groups using anti-HSP-70, and the sections were examined at × 400 magnification. Control animals exhibited normal distribution of HSP-70-secreting lymphocytes (brown color) in the periarterial lymphatic sheath of white pulp in spleen sections (Fig. 5M). However, diabetic animals exhibited a marked increase in the distribution of HSP-70-secreting lymphocytes in the periarterial lymphatic sheath of the white pulp in spleen sections (Fig. 5N). In Diab.+BG animals, the distribution of lymphocytes expressing HSP-70 in the periarterial lymphatic sheath of white pulp in spleen sections was partially restored, similar to that in the control group (Fig. 5O). Moreover, we investigated the effect of diabetes on the phosphorylation of AKT (a promoter of survival) in spleen sections in the three groups using anti-phospho-AKT (p-AKT). The spleen sections showed normal expression of phospho-AKT in the lymphatic sheath of white pulp in control mice (Fig. 5P), while the diabetic group exhibited decreased expression of phospho-AKT in the lymphatic sheath of white pulp (Fig. 5Q). BG-treated diabetic mice exhibited partial restoration in the expression of phospho-AKT in the lymphatic sheath of white pulp similar to that observed in control mice (Fig. 5R).

### BG reduces histopathological alterations and enhances the distribution of T and B cells, the expression level of MCP-1, and the phosphorylation level of AKT in the lymph nodes of diabetic mice

**Fig. 6:**
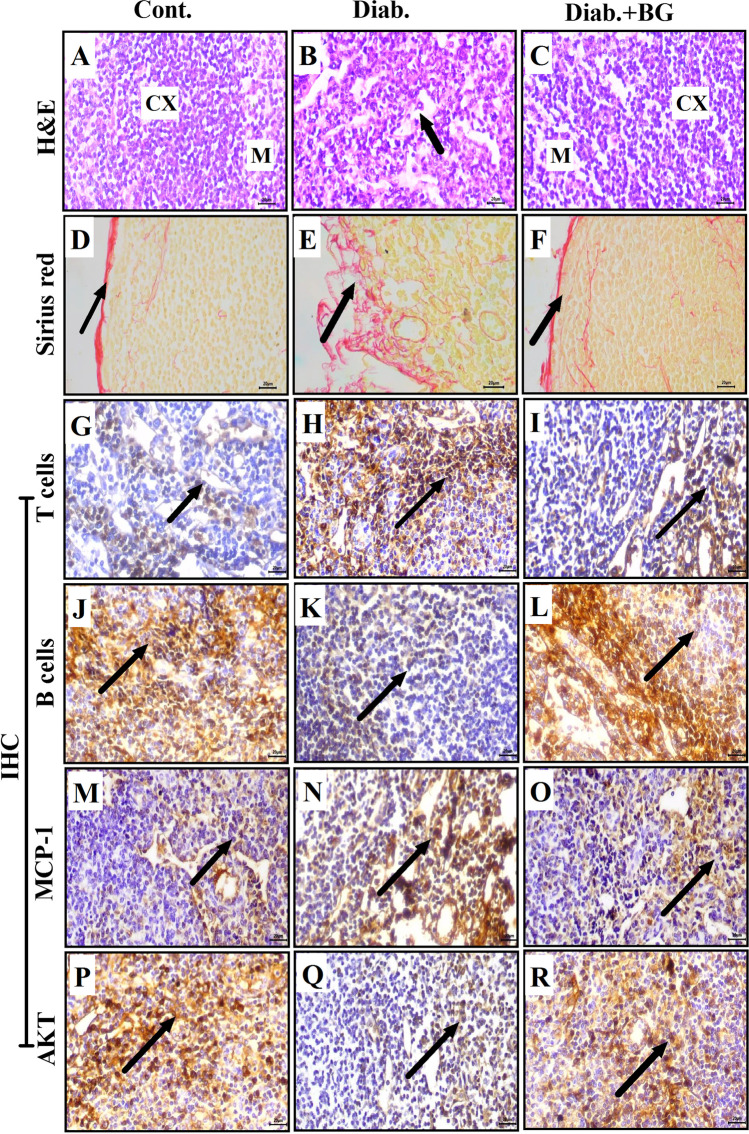
Impact of BG supplementation on the distribution of T and B cells and on the expression of MCP-1 and the phosphorylation of AKT in the lymph nodes of diabetic mice. Lymph node sections from the three groups were assessed by H&E, Sirius red, and immunohistochemical staining. Photomicrographs of lymph node sections from the negative cont., diab., and diab.+BG groups were stained with H&E to examine pathological changes in the architecture of lymph nodes (**A–C**) and Sirius red to examine collagen deposition (**D–F**) and were assessed by immunohistochemistry with anti-CD4 (**G–I**), anti-CD19 (**J–L**), anti-MCP-1 (**M–O**), and anti-AKT (**P–R**). (× 400)

We examined the effect of BG on the lymph nodes (secondary immune organs) in the three groups after the induction of diabetes using H&E, Sirius red, and IHC staining. H&E staining was performed; a photomicrograph was taken at × 400 magnification in the control, diabetic, and Diab.+BG groups; and one representative image from each group is shown. Lymph node sections from control animals exhibited a normal histological appearance of the cortex and medulla (Fig. 6A). However, lymph node sections in the diabetic group demonstrated degenerative changes, as indicated by the depletion of lymphocytes in the cortex and medulla (Fig. 6B). The Diab.+BG group showed a normal histological appearance in the cortex and medulla compared to that in the diabetic group (Fig. 6C). Sirius red staining was performed, and photomicrographs of lymph node sections in the three groups were taken at × 400 magnification. Our results revealed that the control group exhibited normal collagenous fiber deposition in the capsule (Fig. 6D). Diabetic animals exhibited a marked increase in the deposition of collagenous fibers in the capsule (Fig. 6E). Most interestingly, treatment of the diabetic group with BG restored the amount of collagenous fiber deposition in the capsule to nearly that in the control group (Fig. 6F). IHC staining was performed, and we then investigated the distribution of T and B cells in the lymph node at × 400 magnification using anti-CD4 and anti-CD19 antibodies. The lymph node sections exhibited normal numbers of T cells in the sheaths of lymphoid follicles in control mice (Fig. 6G), while diabetic mice exhibited an increase in the number of T cells in the sheaths of lymphoid follicles (Fig. 6H). BG-treated diabetic mice showed partially restored numbers of T cells in the sheath of lymphoid follicle similar to that observed in control mice (Fig. 6I). Additionally, control mice exhibited normal numbers of B cells in lymphoid follicles in lymph node sections (Fig. 6J), whereas diabetic animals exhibited decreased numbers of B cells in lymphoid follicles (Fig. 6K). Supplementation of diabetic mice with BG partially restored the distribution of B cells in the lymphoid follicle to nearly that of control mice (Fig. 6L). Additionally, the expression of MCP-1 in lymph node sections was examined. Normal expression of MCP-1 in the lymphatic sheath of lymphoid follicles was investigated in control mice (Fig. 6M), while the diabetic group exhibited a marked increase in the expression of MCP-1 (Fig. 6N). Treatment of diabetic mice with BG showed partial restoration in the expression of MCP-1 (Fig. 6O). Furthermore, we investigated the expression level of phospho-AKT in lymph node sections in the three groups. The lymph node sections showed normal expression of phospho-AKT in the lymphatic sheath of lymphoid follicles in control mice (Fig. 6P), while the diabetic group exhibited a decrease in the expression level of phospho-AKT (Fig. 6Q). BG-treated diabetic mice exhibited partial restoration in the expression of phospho-AKT (Fig. 6R).

### BG repairs the architecture of lymphoid organs by restoring the expression of autophagy proteins (LC3, Beclin-1 and P62) and apoptotic proteins (Bcl-2 and Bax) in diabetic animals

**Fig. 7:**
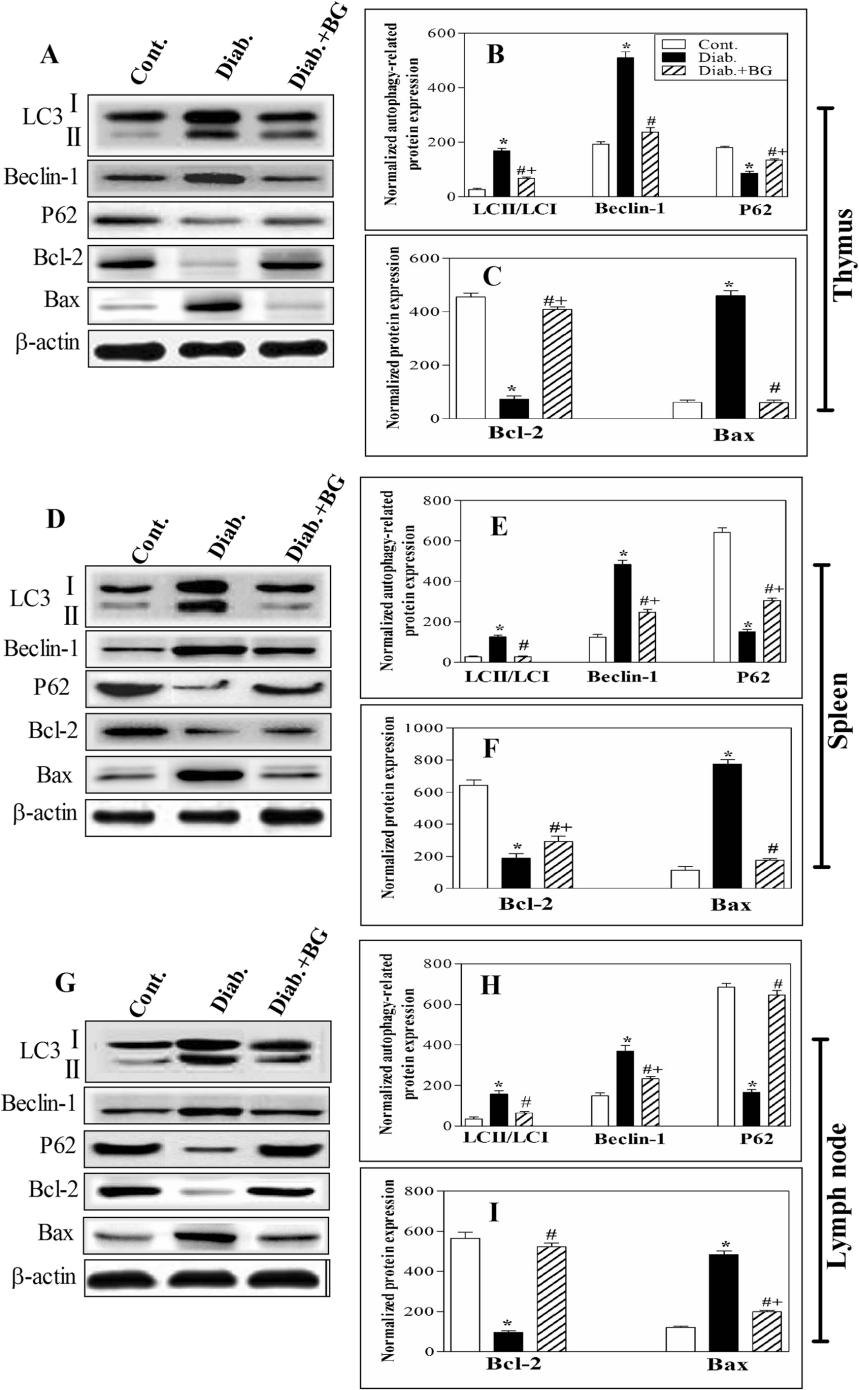
Impact of BG supplementation on the expression of autophagy and apoptotic indicators in the thymus, spleen, and lymph nodes of diabetic mice. **A** Representative immunoblots showing LC3, Beclin-1, P62, Bcl-2, Bax, and β-actin in the thymus in cont., diab., and diab.+BG mice. **B** Quantification of normalized LC3, Beclin-1, and P62 and **C** quantification of normalized Bcl-2 and Bax protein expression levels relative to total actin were determined by western blotting. **D** Representative immunoblots showing LC3, Beclin-1, P62, Bcl-2, Bax, and β-actin in the spleen in the cont., diab., and diab.+BG groups. **E** Quantification of normalized LC3, Beclin-1, and P62 and **F** the quantification of normalized Bcl-2 and Bax protein expression levels relative to total actin were determined by western blotting. **G** Representative immunoblots showing LC3, Beclin-1, P62, Bcl-2, Bax, and β-actin in the lymph nodes in the cont., diab., and diab.+BG groups. **H** Quantification of normalized LC3, Beclin-1, and P62 and **I** the quantification of normalized Bcl-2 and Bax protein expression levels relative to total actin were determined by western blotting. cont. (open bars), diab. (closed black bars), and treated diab.+BG animals (hatched bars). The data are expressed as the means ± SEM (*n* = 5 per group) and were analyzed by ANOVA with Tukey’s post hoc test. Differences were considered statistically significant at *P < 0.05 for diab. vs. cont.; +*P* < 0.05 for diab.+BG vs. cont.; and #*P* < 0.05 diab.+BG vs. diab. group

We assessed the expression of autophagy proteins (LC3, Beclin-1 and P62) and apoptotic proteins (the anti-apoptotic Bcl-2 and pro-apoptotic Bax) in thymus, spleen, and lymph node tissues in the three animal groups using western blot analysis. Fig. 7 shows immunoblots of autophagy proteins (LC3, Beclin-1, and P62), apoptotic proteins (Bcl-2 and Bax), and β-actin (loading control) in thymus (Fig. 7A), spleen (Fig. 7D), and lymph node (Fig. 7G) lysates from control, diabetic, and Diab.+BG mice. The expression levels of autophagy proteins (Fig. 7B, E, and H) and apoptotic proteins (Fig. 7C, F, and I) were normalized to the expression level of total β-actin, and the data from five individual mice are presented as the means ± SEM of the normalized values in the three immune organs. Our data revealed that diabetic mice exhibited significant upregulation in the expression of LC3 and Beclin-1 and downregulation in the expression of P62 compared to control mice. When diabetic mice were orally supplemented with BG, marked reductions in the expression of LC3 and Beclin-1 and increased expression of P62 to nearly that of control mice were observed. Additionally, our results demonstrated that diabetic mice exhibited significant upregulation in the expression of Bax and downregulation in the expression of Bcl-2 compared to control mice. When diabetic mice were treated with BG, the expression levels of Bax and Bcl-2 in the thymus, spleen, and lymph nodes were significantly restored to nearly the levels of the control mice.

## Discussion

T1D is characterized by the production of autoantibodies and the progressive infiltration of immune cells into the islets of the pancreas, followed by the destruction of pancreatic β cells (Van Belle et al. 2011). Immune system impairment in diabetic animals is associated with an increase in blood glucose levels and a decrease in body weight, and these effects are partially restored by BG treatment. Similarly, camel whey protein (CWP) treatment decreased blood glucose levels in T1D (Ebaid et al. 2013). It has been previously demonstrated that the induction of diabetes in animal models was associated with several complications, including elevated oxidative stress markers and proinflammatory signals (Al Ghamdi et al. 2015; Badr 2013). In this context, these results are consistent with our current results, which demonstrated that IL-6 and ROS levels were significantly increased in diabetic mice. Additionally, we found decreases in the levels of IL-2 and IL-7 (cytokines that act as survival factors for T cells and are important for cellular immunity) and in the levels of IL-4 (a cytokine that is required for the activation of B cells and is important for humoral immunity), suggesting a reduction in adaptive immunity in diabetic mice. When the diabetic mice were treated with BG, the levels of IL-2, IL-7, and IL-4 were significantly restored, suggesting the therapeutic potential of BG for improving adaptive immunity. Increased ROS during diabetes can damage cellular components such as lipids, proteins, and DNA (Golbidi and Laher 2010). Moreover, increased inflammation and the development of vascular disease and atherosclerosis are associated with elevated levels of IL-6 in diabetic individuals (Roy and Rosas 2021). Additionally, it has been established that the induction of diabetes in animal models is associated with perturbations in the levels of different cytokines, including IL-2, IL-7, and IL-4, which can perturb adaptive immunity. Moreover, decreased production of IL-2 and IL-7 leads to a decrease in the number and function of regulatory T cells (Tregs), which are responsible for cellular immunity (Bhadra et al. 2010; Geng et al. 2012). Furthermore, decreased production of IL-4 leads to perturbations in the activation and function of B cells, and hence leads to defects in humoral immunity (Erbay et al. 2018). In our study, we found that the induction of diabetes was associated with decreased levels of IL-2, IL-7, and IL-4, which reflects perturbations in adaptive immunity. Interestingly, treatment of diabetic mice with BG restored the levels of IL-2, IL-7, and IL-4 and subsequently enhanced adaptive immunity. Similarly, Al Ghamdi et al. (2015) showed that propolis supplementation restored the levels of IL-2, IL-4, and IL-7 in a diabetic mouse model.

GSH is an intracellular antioxidant that protects against oxidation because its sulfhydryl group is a strong nucleophile that protects DNA, proteins, and other biomolecules from ROS (Forman et al. 2009). MnSOD and GPx-1 are the primary mitochondrial antioxidant defense systems because of their localization in the mitochondrial matrix, which is close to the site of ROS production by the electron transport chain. MnSOD, GSH Px, and catalase clear intracellular superoxide radicals by converting them into water (Wang et al. 2011). Therefore, we established in the current study that diabetic mice exhibited significant reductions in the levels of GSH and the activities of MnSOD, GSH Px, and catalase in plasma compared to control non-diabetic mice.

These defects in antioxidant enzymes contribute to histopathological changes in the structures of immune organs (Moreno et al. 2011). Therefore, our findings showed that BG plays a direct role in improving GSH levels and antioxidant enzyme activity in diabetic animals.

Uncontrolled hyperglycemia can disrupt the structure and function of lymphoid organs (Giri et al. 2018). In this study, the induction of diabetes affected lymphoid organs and caused degenerative changes such as necrosis, hemorrhage, congestion of the blood vessels, and the depletion of lymphocytes and fibrosis, which mediated dysfunction in these lymphoid organs and was consistent with the results of previous studies (Aktuğ et al. 2010; Qinna and Badwan 2015; Tuleta and Frangogiannis, 2021; Udumula et al. 2021). However, treatment of diabetic animals with BG improved the histological architecture of lymphoid organs and reduced fibrosis, resulting in improvements in the immune functions of lymphoid organs. Subsequently, this effect will facilitate overcoming the complications associated with diabetes. Likewise, melatonin treatment protects lymphoid organs from diabetic effects (Ozkanlar et al. 2016). Similarly, Sayed et al. (2021) observed that propolis supplementation decreased collagenous fibers in the lymphoid organs of CCl4-treated animals.

In this study, we found that T-cell infiltration was increased in the lymphoid organs of diabetic animals and disrupted cellular immunity. Treatment with BG facilitated the release of mature T cells into the periphery, which led to improvements in the immune response. The number of B cells was decreased in diabetic animals in our current study, which led to disturbances in humoral immunity. Moreover, a decrease in the number of B cells in diabetic mice results from a decrease in the level of IL-4, which plays an important role in the function and activation of B cells (Erbay et al. 2018). Treatment with BG restored the number of B cells in the diabetic mouse model. Similarly, WP treatment restored the number of T and B cells in diabetic mice (Ebaid 2014).

HSP-70 (a marker of inflammation) regulates intracellular protein homeostasis and prevents toxic aggregate formation, which leads to inflammation or cell death (Leite et al. 2016). MCP-1 is a crucial chemokine that regulates monocyte/macrophage migration (Deshmane et al. 2010). The increased levels of HSP-70 and MCP-1 in diabetes are positively associated with markers of inflammation (Nakhjavani et al. 2010; Soetikno et al. 2011), which is in agreement with our findings. Most importantly, BG treatment restored the expression of HSP-70 and MCP-1 in the lymphoid organs of diabetic animals, subsequently decreasing inflammation and enhancing the immune functions of lymphoid organs.

Additionally, Akt belongs to a family of serine-threonine protein kinases that regulates cell survival and protects cells against apoptosis (Androulidaki et al. 2009; Engedal 2011). Our current study showed downregulation in AKT phosphorylation in the lymphoid organs of diabetic animals compared to the upregulation that occurred in the context of BG treatment.

Both apoptosis and autophagy are important in development, normal physiology, and diseases (Thorburn 2008). Autophagy is responsible for the degradation and removal of damaged/long-lived organelles and proteins; thus, a decline in autophagy with age has been associated with a variety of aging disorders, including diabetes (Parzych and Klionsky 2014). The crosstalk between autophagy and apoptosis occurs during cell death (Lin et al. 2014), and the interaction between Bcl-2 and Beclin-1 is necessary to regulate the crosstalk between autophagy and apoptosis (Marquez and Xu 2012; Salminen et al. 2013). In this study, we investigated the expression levels of both Bcl-2 and Beclin-1. The findings of this study are in accordance with previous studies that demonstrated increased expression of Beclin-1 during autophagy induction and decreased expression of Bcl-2 during apoptosis induction (Erlich et al. 2007; Chipuk et al. 2010; Luo and Rubinsztein 2010). Autophagy proteins such as P62, Beclin-1, and LC3 (which are required for autophagosome biogenesis/maturation) regulate selective autophagy, cell survival, cell death, oxidative stress, DNA repair, and inflammation and play important roles in several diseases, such as diabetes and obesity (Guan et al. 2016; Lee and Lee 2016; Fan et al. 2018). The present study revealed that upregulated expression of LC3 and Beclin-1 and downregulated expression of P62 in lymphoid tissues of diabetic mice led to the dysregulation of autophagy, and these results are consistent with the findings of a previous study (Kong et al. 2018). Interestingly, treatment of diabetic mice with BG restored the expression of LC3, Beclin-1, and P62 to levels similar to those found in control mice.

Additionally, the Bcl-2 family is found on the membranes of mitochondria and functions as anti- or proapoptotic regulators. Bcl-2 is an antiapoptotic factor that can maintain apoptogenic factors in mitochondria, whereas Bax, a proapoptotic regulator, enhances the release of those factors (Liu et al. 2011). Moreover, we found that the expression of Bcl-2 (antiapoptotic) decreased and Bax (proapoptotic) increased in the lymphoid organs of diabetic mice, which led to increased apoptosis, which was consistent with He et al. (2018). Interestingly, BG treatment restored the expression of Bcl-2 and Bax in diabetic mice and regulated apoptosis.

Because diabetic complications are life-threatening, new strategies should be applied to reduce the side effects of diabetic complications. Since BG reduces oxidative stress and inflammatory markers, repairs the architecture of lymphoid organs, and regulates apoptosis and autophagy without any side effects, it could be a new agent to overcome diabetic complications.

## Conclusion

There are no published data on the biological effects of BG on diabetes and its associated complications. Hence, in the present work, oral supplementation with BG exerted therapeutic effects on diabetic mice by restoring blood glucose levels, reducing free radical levels (ROS), restoring inflammatory markers, and enhancing the activities of antioxidant enzymes (MnSOD, GSH Px, and catalase). Additionally, BG regulated the expression of autophagy- and apoptosis-related proteins, which repaired the histopathological features induced by T1D in lymphoid organs. Our study was the first to show that treatment of diabetic mice with BG decreased diabetic complications in immune organs and improved adaptive immunity by restoring the levels of IL-2, IL-7 (T-cell survival factors), and IL-4 (B-cell activating factor). According to these results, we investigated the therapeutic potential of BG on the healing process of diabetic wounds (as one of the serious health complications of diabetes) in a mouse model.

## Data Availability

All data are available upon request from the corresponding author (Gamal Badr: badr73@yahoo.com and gamal.badr@aun.edu.eg) as bee gomogenat extraction and preparation
